# Multimodal deep learning model for prediction of prognosis in central nervous system inflammation

**DOI:** 10.1093/braincomms/fcaf179

**Published:** 2025-05-09

**Authors:** Bo Kyu Choi, Yoonhyeok Choi, Sooyoung Jang, Woo-Seok Ha, Soomi Cho, Kimoon Chang, Beomseok Sohn, Kyung Min Kim, Yu Rang Park

**Affiliations:** Department of Biomedical Systems Informatics, Yonsei University College of Medicine, Seoul 03722, Republic of Korea; Department of Neurology, Gangnam Severance Hospital, Yonsei University College of Medicine, Seoul 06273, Republic of Korea; Department of Biomedical Systems Informatics, Yonsei University College of Medicine, Seoul 03722, Republic of Korea; Department of Computer Science and Engineering, Yonsei University College of Computing, Seoul 03722, Republic of Korea; Department of Biomedical Systems Informatics, Yonsei University College of Medicine, Seoul 03722, Republic of Korea; Department of Neurology, Yonsei University College of Medicine, Seoul 03722, Republic of Korea; Department of Neurology, Yonsei University College of Medicine, Seoul 03722, Republic of Korea; Department of Neurology, Uijeongbu Eulji Medical Center, Eulji University, Uijeongbu 11759, Republic of Korea; Department of Radiology, Samsung Medical Center, Sungkyunkwan University School of Medicine, Seoul 06351, Republic of Korea; Department of Neurology, Yonsei University College of Medicine, Seoul 03722, Republic of Korea; Department of Biomedical Systems Informatics, Yonsei University College of Medicine, Seoul 03722, Republic of Korea

**Keywords:** central nervous system inflammation, artificial intelligence, brain MRI, multimodal deep learning

## Abstract

Inflammatory diseases of the CNS impose a substantial disease burden, necessitating prompt and appropriate prognosis prediction. We developed a multimodal deep learning model integrating clinical features and brain MRI data to enhance early prognosis prediction of CNS inflammation. This retrospective study used thin-cut T1-weighted brain MRI scans and the clinical variables of patients with CNS inflammation who were admitted to a tertiary referral hospital between January 2010 and December 2023. Data collected after January 2022 served as the external test set. 3D MRI images were first segmented into 43 brain regions using the FastSurfer library. The segmented images were then processed through a 3D convolutional neural network model for feature extraction and vectorization, after which they were integrated with clinical features for prediction. The performance of each artificial intelligence model was assessed using accuracy, F1 score, area under the receiver operating characteristic curve and area under the precision-recall curve. The internal dataset comprised 413 images from 291 patients (mean age, 45.5 years ± 19.3 [SD]; 151 male patients; 54 with poor prognosis). The external dataset comprised 210 images from 106 patients (mean age, 45.5 years ± 18.9 [SD]; 59 male patients; 31 with poor prognosis). The multimodal deep learning model outperformed unimodal models across all aetiological groups, achieving area under the receiver operating characteristic curve values of 0.8048 for autoimmune, 0.9107 for bacterial, 1.0000 for tuberculosis and 0.9242 for viral infections. Furthermore, artificial intelligence assistance improved clinicians' prognostic accuracy, as demonstrated in comparisons with neurologists, paediatricians and radiologists. Our findings demonstrate that the multimodal deep learning model enhances artificial intelligence-assisted prognosis prediction in CNS inflammation, improving both model performance and clinician decision-making.

## Graphical Abstract

**Graphical Abstract:**
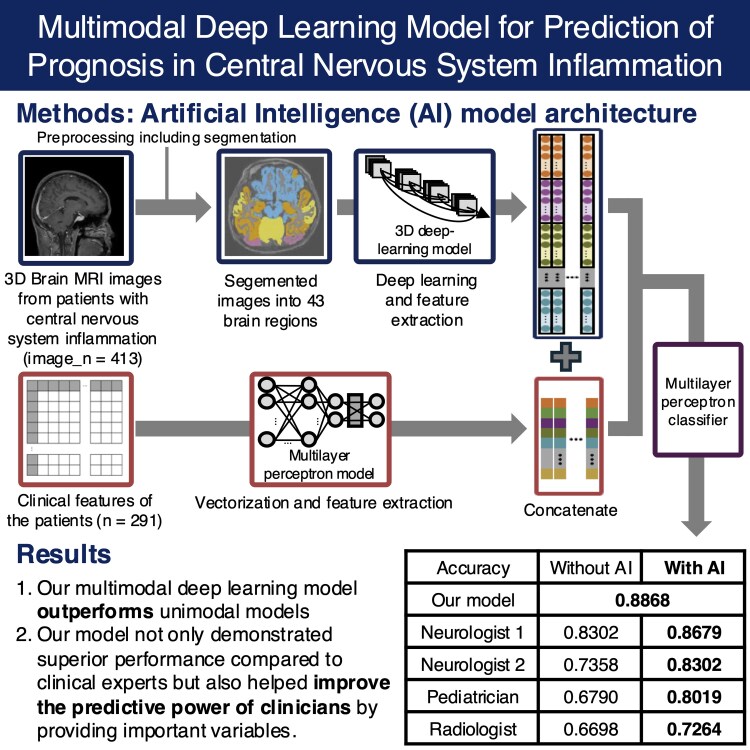

## Teaser

Choi et al. report that a multimodal deep learning model was developed to predict the prognosis of CNS inflammation using brain MRI and clinical data. The model outperformed unimodal models and clinical experts in predicting outcomes across different aetiologies, enhancing artificial intelligence-supported clinical decision-making for CNS inflammation.

## Introduction

The CNS, which comprises the brain and spinal cord, usually is a sterile environment. Inflammation of the CNS due to various aetiologies represents a significant disease burden, often resulting in severe sequelae.^1^ Even patients with mild initial symptoms may experience aggravation during hospitalization, including altered consciousness and focal neurological deficits.^2^ Prompt intervention in worsening cases is crucial for improving prognosis and early prediction of the prognosis of CNS inflammatory diseases could facilitate the provision of intensive care to patient groups with anticipated poor outcomes, ultimately contributing to overall prognosis improvement.^3^ In conventional clinical practice, prognosis is often estimated based on the severity of initial symptoms and laboratory test results, with clinicians potentially relying on their experience.^3^ Previous studies have attempted to predict the prognosis of CNS inflammation, but most are limited by focusing on a single pathogen or specific situations, making their application in clinical practice challenging.^4-6^ For instance, Xiang *et al*.^4^ reported a successful prognostic model, but it was restricted to anti-N-methyl-D-aspartate receptor encephalitis, a single autoantibody-mediated encephalitis, with considerations for future expansion to other subtypes. Similarly, Lu *et al*.^6^ attempted to predict prognosis in patients with severe traumatic brain injury after craniotomy under the specific condition of infection, employing conventional machine learning techniques.

Brain MRI scans are crucial diagnostic tools used in various neurological disorders, and they are also performed to differentiate the causes and assess the severity of CNS inflammation. Distinctive MRI findings based on the underlying aetiologies of CNS inflammation have been identified in previous research.^7^ Furthermore, there are several deep learning studies utilizing brain MRI data to predict the aetiology and prognosis of CNS disorders including neurodegenerative diseases and brain tumors.^8,9^ Recently, there has been a surge in multimodal deep learning (MMDL) research, under the premise that artificial intelligence (AI) models should be able to utilize data from various sources commonly available in clinical practice.^10-12^ Wang *et al*.^11^ reported an AI-enabled cardiac MRI interpretation MMDL model that achieved high diagnostic performance in CVD screening and diagnosis. Wu *et al*.^12^ suggested a MMDL model using preoperative MRI to noninvasively predict lymph node metastasis in cervical cancer demonstrating prognostic value for disease-free survival. It has also been reported that combining brain MRI with clinical variables in autoimmune encephalitis shows higher performance in prognostic prediction compared to using each single modality alone.^4^

In this study, we aim to develop a deep learning model for the early prognosis prediction of CNS inflammation with multimodal data including clinical features and brain imaging data. Furthermore, we validated the potential of AI-assisted diagnosis by demonstrating how our model can not only enhance predictive performance beyond that of experts but also support their clinical decision-making.

## Materials and methods

### Patient selection

Patients diagnosed with encephalitis or meningitis and admitted to Severance Hospital, an academic tertiary care medical centre in South Korea, between 1 January 2010 and 31 December 2023, were recruited retrospectively. Data were collected from the Severance Clinical Research Analysis Portal, which offered anonymized patient data from Severance Hospital to researchers for privacy preservation. Patients over the age of 18 were recruited based on ICD-10 codes indicating diagnosis of encephalitis or meningitis at discharge, and those without cerebrospinal fluid (CSF) analysis results or 3D T1-weighted brain MRI images were excluded. Patients admitted before 31 December 2020, were classified into the internal dataset, while those admitted thereafter were classified into the external validation dataset (Supplementary Fig. 1). Based on the modified Rankin Scale (mRS) score at discharge, all patients were classified into good prognostic groups (mRS scores of 0, 1 or 2) and poor prognostic groups (mRS scores of three or above).

### Data pre-processing

The sagittal sections of the brain MRI images with Digital Imaging and Communications in Medicine (DICOM) format were converted to 3D Neuroimaging Informatics Technology Initiative (NIfTI) images with Python dicom2nifti library. The NIfTI images were then resampled by lab2im library which resized the images and set the voxel resolutions to 1 mm^3^.^13^ The resampled images with uniform size were then conformed to the following specifications: linear min–max intensity normalization for adherence to the Unsigned Character (UCHAR) format (0∼255), an image size of 256 × 256 × 256, isotropic voxel dimensions ranging from 1 to 0.7 mm and a standard slice orientation of left, inferior, and anterior with FastSurfer library.^14^ Intensities in the images are inhomogenous and this issue affects the performance of the method. To cope with this issue, an intensity normalization process is applied. Although various normalization algorithms with different image types have been implemented in the literature, they may lead to high computational costs. Therefore, in the proposed approach, intensity values in the MRI images have been normalized with linear min–max intensity normalization. After conformation task, the images were segmented into 48 brain regions with FastSurfer v2.2 library. Neurologists and neuroradiologists manually verified the segmentation by checking the mean volume and shape of each brain subregion. A total of 43 brain regions were used to train the deep learning model, excluding the ventricular area. The schematic diagram of the pre-processing pipeline is provided in Supplementary Fig. 2. Clinical features including demographic characteristics, vital signs and laboratory findings within initial 24 h were collected. In cases where there were multiple instances of laboratory findings for a patient, the initial values were utilized. For vital signs, the average values were used. All numerical variables were normalized with min–max scaling and missing values were replaced with −1. Based on the multicollinearity among variables and their clinical importance, a total of 68 clinical variables were used.

### Unimodal model training

To mitigate potential bias in prognostic predictions caused by aetiological differences, patients were divided into four aetiological groups: autoimmunity, bacteria, tuberculosis and virus. Patients of each aetiology were split into five subgroups, with an even age distribution within each subgroup considering for age-related variations in brain volume. Among the five subgroups, one was used as the fixed internal held-out test set and the others were used to train the models using k-fold cross validation. To eliminate bias, multiple brain MRI data obtained from the same patient were grouped into the same fold and utilized for training. Since the brain is inherently symmetrical and there is no strong evidence suggesting a predilection for CNS inflammation on either the left or right side, brain regions present in both hemispheres were combined and used for training.

A modified denseNet-169 based deep learning model for brain MRI images from each brain part of each aetiology were built.^15^ The training was performed with 4-fold cross validation and the model with the best performance was chosen for the inference task (Supplementary Fig. 3). In consideration of multimodal deep learning, clinical variables were vectorized at patient-level. They were then trained using a multilayer perceptron (MLP) architecture. The train/test validation set was constructed same to the MRI dataset. Data imbalance between the outcome was handled by weighted random sampling. The performance of each unimodal model was assessed using the area under the receiver operating characteristic curve (AUROC). To check the interpretability of the unimodal model using brain MRI data, hierarchical clustering method was used based on the prediction results. This process aimed to verify whether adjacent brain regions exhibited similar performance.

### Feature selection

To ensure systematic application and better comprehension, we implemented a feature selection method separately for the clinical and MRI datasets. The Shapley Additive exPlanations (SHAP) values were used to derive feature importance and explain the model.^16^ As SHAP values are model agnostic, it can be used to explain contributions of features across different modalities. We developed a method for selecting important variables applicable to this study based on existing methods such as groupShapley methods for vectorized features.^17^ As the time complexity of direct SHAP calculation is exponential, implementation of an approximation method is mandatory for a large number of features. The approximation method was implemented using coalition vectors^18^ and the details of the approximation algorithm are provided in Appendix 1 of the Supplementary Materials.

### Multimodal model training

**Figure 1:**
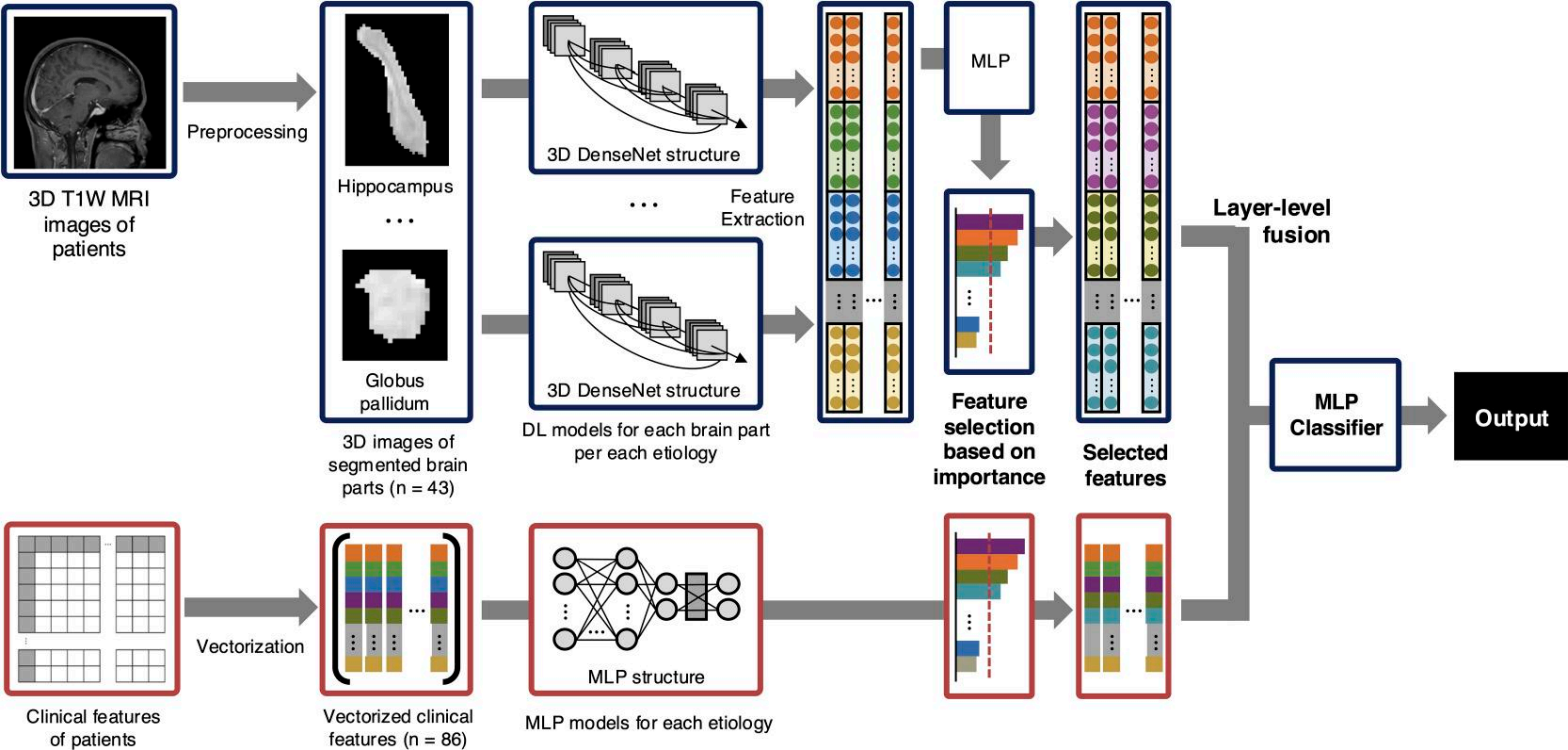
**The schematic diagram of the overall training process for multimodal deep learning.** DL, deep learning; MLP, multilayer perceptron; DenseNet, densely connected convolutional networks.

Clinical and brain imaging variables underwent an independent feature selection method when combined. For the MRI data, vector representations were extracted from each brain subregion through adaptive average pooling, using the MRI unimodal model of itself as a feature extractor. As SHAP values for feature groups are the direct summation of SHAP values for each individual feature in the group, we can group each brain subregion into groups, and then divide the SHAP value of the group by the size of the group to obtain the mean contribution of the brain regions in the group to the model output. Each brain subregion was therefore grouped with anatomical knowledge to minimize multicollinearity between brain subregions,^17^ and SHAP values evaluated on the all-features MLP model were used to measure the importance for each group. Based on feature importance, the top 10 of 14 groups were selected. Clinical features were also vectorized and their SHAP values from the MLP model were used to measure the importance for each group. Due to the relatively smaller size of the clinical variable vector compared to the brain imaging, the top 30 of 68 features were selected. The extracted data were then concatenated for multimodal deep learning training. The combined multimodality data vector was fed to a MLP classifier for the final prognosis prediction. Model explainability was analysed using SHAP values for each feature on the final classifier. The schematic diagram of the overall training process for MMDL is presented in Fig. 1. The hyperparameters for each model are provided in Appendix 2 of the Supplementary Materials. The performance of MMDL model was assessed using accuracy, F1 score, AUROC, and the area under the precision-recall curve (AUPRC).

### AI explainability

After performing MMDL, we also calculated SHAP values as previously described to ensure model interpretability. For further explainability on our multimodal models which will be described later, supervised clustering based on SHAP values was performed to convert the units of the input features to the same units as the model output regardless of the original unit. This ensures that the changes in the feature values are comparable regardless of feature characteristics and effect the cluster formation only if the changes have any meaningful impact on the final outcome. Uniform Manifold Approximation and Projection for Dimension Reduction (UMAP) was used to reduce dimensionality and boost the performance of density-based clustering while preserving the overall structure of the data. The density-based spatial clustering of applications with noise (DBSCAN) algorithm was used to cluster the data in the reduced dimensions.^19^

### Clinical comparison

To evaluate the potential clinical utility of the AI model, four clinicians were recruited, including one neuroradiologist, one paediatrician, and two neurologists. For testing purposes, the initial brain MRI scans of all 106 patients used in the external validation were provided. Initially, we provided the doctors with NIfTI-format 3D T1-weighted MRI images and tabular clinical variables to predict the prognosis of each patient. A week later, we presented the same data along with the predictions from each AI model to assess whether the prediction performance of the clinicians improved.

### Statistical analysis

All numerical variables were normalized with min–max scaling and missing values were replaced with −1. Hierarchical clustering was performed using an agglomerative approach with average linkage. Euclidean distance was used as the dissimilarity metric, and the optimal number of clusters was set to *k* = 6 based on the silhouette method, selecting the number of clusters that maximized the silhouette coefficient.

For efficient SHAP estimation, we employed a batch-sampling strategy, selecting one-fifth of the test dataset at a time, randomly, to compute SHAP values for each instance. A single coalition was applied per batch. During this process, two tensors were generated: one representing the original dataset and the other representing the sampled dataset. Each tensor had dimensions corresponding to the number of sampled instances and the size of the feature vector, ensuring that the SHAP estimation was applied consistently across all selected data points. This implementation, combined with parallelized models, imposed substantial memory overhead. As in the original SHAP framework, we imposed a maximum error tolerance between the summation of model predictions and the summation of SHAP values, ensuring local accuracy (Appendix 1 of the Supplementary Materials).

### Computational hardware and software

We processed all MRIs and clinical data on a computing workstation with Intel Xeon Gold 6326 CPU 2.90 GHz 16 core processor, and 2 NVIDIA A40 46GB GPUs. The pre-processing of clinical variables and hierarchical clustering process were conducted using R version 4.11. The pre-processing of brain MRI and the entire deep learning process were performed using Python version 3.10.4. Each deep learning model was developed using PyTorch version 1.13.1.

### Ethics

This study was approved by the Yonsei University Health System, Institutional Review Board (Y-2021-0960). Due to the retrospective nature and use of de-identified data, this study was approved with waiver of the requirement to obtain informed consent by the Yonsei University Health System, Institutional Review Board (Y-2021-0960). The study was performed in accordance with approved guidelines and regulations for medical research expressed in the Declaration of Helsinki.

## Results

### Patient characteristics

Between 1 January 2010, and 31 December 2020, patients with CNS inflammation (*n* = 291) were used for model training. Virus was the most common aetiology (*n* = 170), followed by bacteria (*n* = 46), autoimmunity (*n* = 45) and tuberculosis (*n* = 30). Patients who were first diagnosed with CNS infection and admitted between 1 January 2021 and 31 December 2023 (*n* = 106), were used as an external validation dataset. Multiple brain MRI scans taken during hospitalization for each patient were included, resulting in a total of 413 objects in the internal training dataset and 211 objects in the external validation dataset. The baseline characteristics according to different aetiology are presented in Supplementary Table 1. As reported in previous literature, differences in clinical variables, including CSF test results, were observed depending on the aetiology. Characteristically, it was observed that the prognosis is relatively good among patient groups with viral infections.

### Unimodal model performance

**Figure 2:**
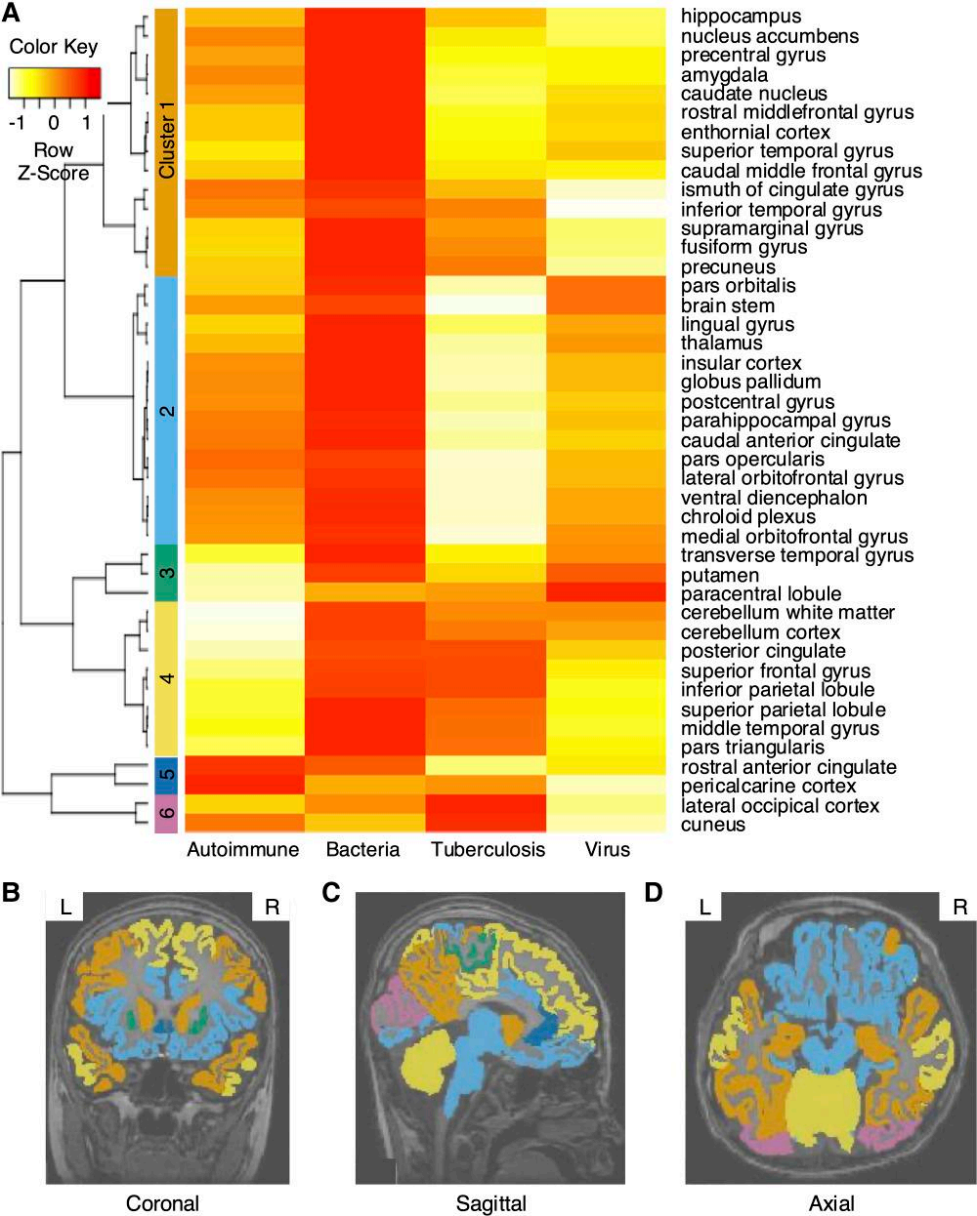
**Hierarchical clustering results based on predictive performance of unimodal models using brain MRI.** (**A**) The 43 brain regions were divided into 6 clusters. (**B–D**) Visualization of hierarchical clustering of each brain region overlaid on brain MRI images. (**B**) Coronal, (**C**) sagittal and (**D**) axial section. Hierarchical clustering was performed using an agglomerative approach with average linkage and Euclidean distance as the dissimilarity metric. Clustering was conducted on an internal dataset derived from the results of the MRI unimodal model. The dataset included autoimmune (*n* = 45), bacterial (*n* = 46), tuberculosis (*n* = 30) and viral (*n* = 170) cases.

For each brain part of each aetiology, the 3D image dataset was used to develop prognostic models for prediction. The prognostic performance according to segmented brain regions for each aetiology is presented in the Supplementary Table 2. The brain regions with high predictive performance vary depending on the cause, but there are also regions such as the hippocampus and nucleus accumbens that generally show good predictive performance. Hierarchical clustering resulted in six clusters for a total of 43 brain regions (Fig. 2A). Cluster 1 included cortical regions of the temporal lobe, while cluster 2 comprised subcortical regions associated with the limbic system. Visualizing each cluster overlaid on brain MRI data revealed that similar brain regions were grouped into similar clusters (Fig. 2B–D). The performance of the unimodal model, trained using the feature vectors derived from brain MRI data through a convolution neural networks (CNN) model and further processed through an MLP layer, is presented in Table 1. In terms of AUROC, the MRI-based unimodal model outperformed the clinical feature-based unimodal model in bacterial and viral aetiologies, while the opposite trend was observed for the others.

**Table 1:** Prognostic prediction performance for each aetiology of central nervous system inflammation

Aetiology		Autoimmune (internal_n = 45, external_n = 46)				Bacterial (internal_n = 46, external_n = 8)				Tuberculosis (internal_n = 30, external_n = 3)				Viral (internal_n = 170, external_n = 66)			
Metrics		AUROC	AUPRC	Accuracy	F1 score	AUROC	AUPRC	Accuracy	F1 score	AUROC	AUPRC	Accuracy	F1 score	AUROC	AUPRC	Accuracy	F1 score
Unimodal model with MRI data	Internal	1.0000	1.0000	1.0000	1.0000	1.0000	1.0000	1.0000	1.0000	0.7778	0.8095	0.8889	0.8000	0.7836	0.3912	0.7447	0.5714
	External	0.6604	0.6331	0.6794	0.5762	0.7500	0.8103	0.7333	0.7500	0.7500	0.5000	0.8000	0.6667	0.8720	0.7455	0.8407	0.6667
Unimodal model with clinical dada	Internal	0.6071	0.2576	0.8125	0.4000	1.0000	1.0000	1.0000	1.0000	1.0000	1.0000	1.0000	1.0000	0.8947	0.6765	0.8297	0.6923
	External	0.7393	0.7599	0.7692	0.7000	0.7500	0.7583	0.8667	0.8889	1.0000	1.0000	1.0000	1.0000	0.8382	0.4809	0.7965	0.6667
Multimodal model with MRI and clinical data	Internal	1.0000	1.0000	1.0000	1.0000	1.0000	1.0000	1.0000	1.0000	1.0000	1.0000	1.0000	1.0000	0.8216	0.4485	0.7872	0.6154
	External	0.8048	0.7898	0.7564	0.6885	0.9107	0.9519	0.9333	0.9333	1.0000	1.0000	1.0000	1.0000	0.9242	0.8316	0.9381	0.8372

AUROC, the area under the receiver operating characteristic curve; AUPRC, the area under the precision-recall curve.

### Multimodal model performance

**Figure 3:**
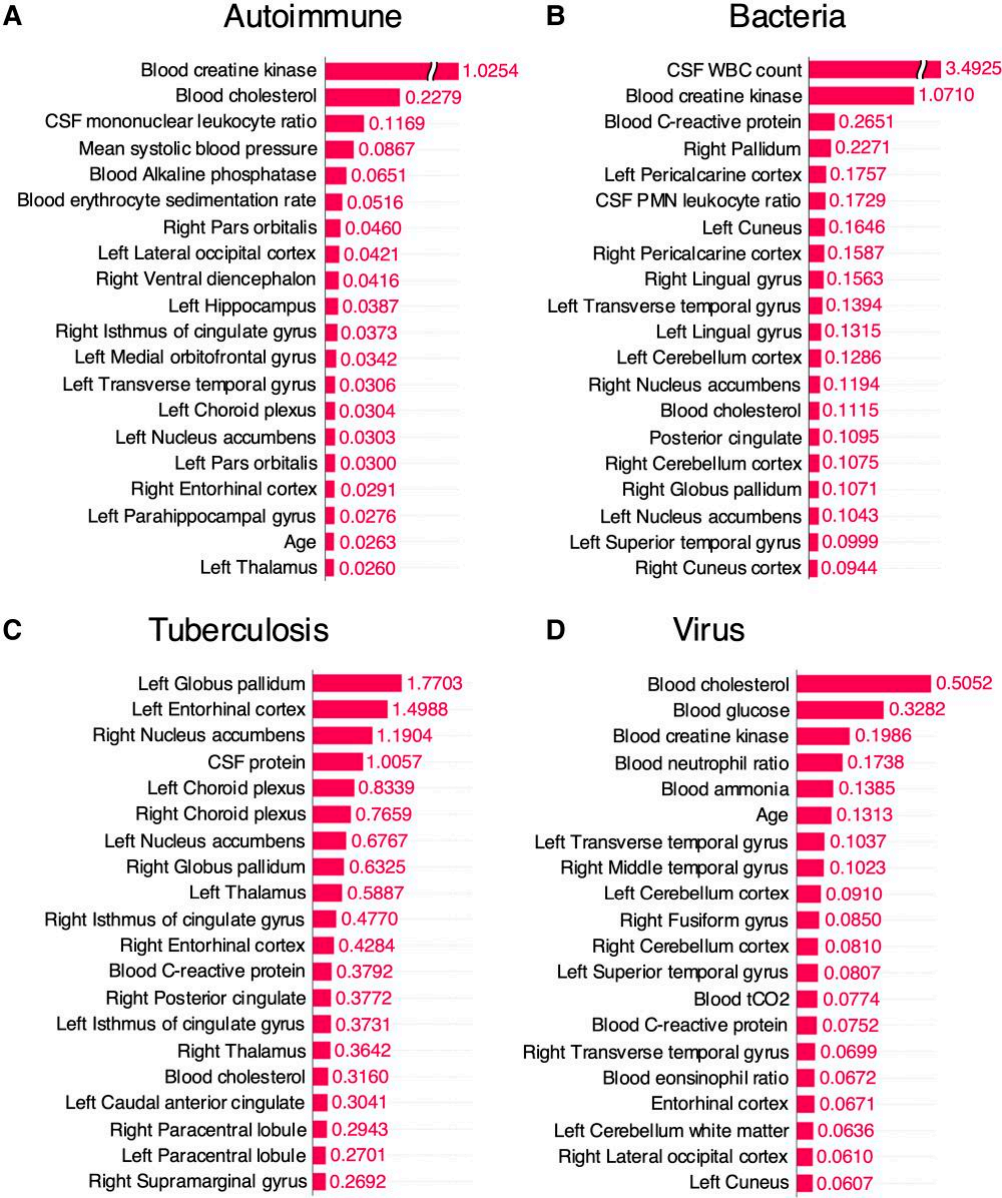
**Top 20 variables crucially utilized in multimodal deep learning for each aetiology in the external test set and their SHAP values.** A batch-sampling strategy was employed for SHAP estimation, selecting one-fifth of the test dataset at a time to compute SHAP values while balancing memory efficiency and estimation performance. (**A**) Autoimmune (*n* = 29), (**B**) bacteria (*n* = 8), (**C**) tuberculosis (*n* = 3) and (**D**) virus (*n* = 66). CSF, cerebrospinal fluid; WBC, white blood cell; PMN, polymorphonuclear.

The multimodal model, which combines clinical and brain imaging variables yielded better performance overall compared to individual models using either clinical or imaging variables alone for prognostic prediction. The selected features from each modality for MMDL are presented in Supplementary Tables 3 and Supplementary Fig. 4. In all aetiological groups, including autoimmune (AUROC = 0.8048), bacterial (AUROC = 0.9107), tuberculosis (AUROC = 1.0000) and viral (AUROC = 0.9242), the models demonstrated predictive power of over 80% on external test set (Table 1). Even when predicting outcomes for all patients without information about the cause of inflammation, we could observe improved performance in the multimodal model compared to the single-modality model (Supplementary Table 4). The SHAP values were computed for each feature vector used in the multimodal model predictions and presented the top 20 variables in Fig. 3 and Supplementary Fig. 5. Since different variables were selected and used for training based on the aetiology, they display differing levels of significance (Supplementary Table 5). Interestingly, clinical and brain imaging variables consistently showed high SHAP values across all aetiologies. Additionally, dimensional reduction of SHAP values with UMAP and DBSCAN algorithm for each feature vectors revealed a tendency for patients with the same mRS score to cluster together (Supplementary Fig. 6).

### Clinician performance

Based on multimodal data, 4 doctors predicted the prognosis of 106 patients (Table 2). The predictive performance of clinicians, measured by accuracy, was lower compared to the MMDL model: radiologists scored 0.6698, paediatricians 0.6790, junior neurologists 0.7358 and senior neurologists 0.8302, while the MMDL model achieved 0.8868. When re-evaluation was performed based on the assistance of the AI model, all clinicians showed improved predictive performance: radiologists scored 0.7264, paediatricians 0.8019, junior neurologists 0.8302 and senior neurologists 0.8679.

**Table 2:** The performance metrics comparing the AI model with clinicians for predicting the prognosis of central nervous system inflammation

		Accuracy	Precision	Recall	F1 score
AI models	Unimodal model with MRI data	0.7830	0.8571	0.8675	0.8623
	Unimodal model with clinical dada	0.7642	0.8919	0.7952	0.8408
	Multimodal model with MRI and clinical data	0.8868	0.9176	0.9398	0.9286
Initial prediction	Junior neurologist	0.7358	0.8571	0.7952	0.8250
	Senior neurologist	0.8302	0.8916	0.8916	0.8916
	Neuroradiologist	0.6698	0.8750	0.6747	0.7619
	Paediatrician	0.6970	0.8077	0.8077	0.8077
AI-supported prediction	Junior neurologist	0.8302	0.9114	0.8675	0.8889
	Senior neurologist	0.8679	0.9157	0.9157	0.9157
	Neuroradiologist	0.7264	0.8649	0.7711	0.8153
	Paediatrician	0.8019	0.8875	0.8554	0.8712

## Discussion

In this retrospective prognostic classification study, we developed a MMDL algorithm capable of predicting CNS inflammation by using brain MRI and clinical information from 291 patients. The performance of the model was evaluated using a separate dataset consisting of 106 patients, demonstrating high accuracy and good discriminative ability. To our knowledge, this study is the first to perform prognostic prediction of CNS inflammatory diseases, regardless of the cause, using a MMDL approach. The algorithm not only demonstrated superior performance compared to clinical experts but also helped improve the predictive power of clinicians by providing important variables.

Prompt and appropriate treatment of CNS inflammation is crucial, especially when the brain is involved, leading to complications such as seizures or altered consciousness, necessitating rapid initiation of intensive care measures such as respiratory support.^2,3^ However, due to economic and human resource constraints, it is necessary to predict and prepare for disease aggravation in advance, as continuous monitoring for all patients is impractical. Previous attempts to predict the prognosis of CNS inflammatory diseases have been limited by their focus on specific situations, making them insufficient for widespread application to real world.^4-6^ Meanwhile, in this study, we developed successful prognostic prediction models for the most common causes of inflammation: viral, bacterial, autoimmune, and tuberculosis. Remarkably, we achieved an AUROC of over 75% even without information on the aetiology.

In this study, single-modality models based on clinical variables alone showed unsatisfactory performance overall. This is similar to our previous study, where we attempted prognosis prediction using only initial clinical variables but failed to achieve satisfactory performance.^20^ Also, Broadley *et al*.^21^ reported a review article analysing studies that predicted the prognosis of autoimmune encephalitis using clinical variables. They highlighted a key limitation in these studies, noting that variations in the definition and analytical approach of clinical variables across studies make result comparisons difficult and limit the ability to fully explain long-term neurological recovery. Inspired by recent advances in deep learning research utilizing multimodal data, and considering the use of diagnostic equipment such as brain MRI, computed tomography and electroencephalography in clinical practice to estimate the prognosis of CNS inflammation patients, we incorporated brain MRI variables in addition to clinical variables.^7,22^ While the model based solely on brain MRI modality did not show high predictive performance, the multimodal model combining both modalities demonstrated good predictive performance. To evaluate the model's performance, we used an external dataset to mitigate the risk of overfitting, and the model showed an AUROC of over 80% for all pathogens. Similar to multimodal models applied in other diseases,^10-12,23^ the model presented in this study outperformed unimodal models, providing evidence for further research combining multiple modalities in CNS inflammatory diseases.

In previous studies utilizing brain MRI, the entire brain was commonly used as a single image for prediction.^4,8,9,24^ This method was mainly applied to neurodegenerative diseases with overall brain atrophy or relatively large and clearly visible brain tumours, aiming to reduce information loss and allow intuitive analysis. However, in CNS inflammation, where inflammation may be localized, subtle changes may occur only in specific brain regions rather than affecting the entire brain.^7^ Therefore, the conventional approach of using the entire brain image as input for training may not be effective. To address this, we utilized segmentation techniques to divide the brain into 43 subregions, allowing us to capture subtle changes in each brain region separately and incorporate them into our model training. The whole segmentation was automatically processed by combining existing segmentation codes, which might make fewer errors compared to manual work. Indeed, after segmentation, we evaluated the mean volume of each brain subregion and compared the shapes of the visualized subregions with those obtained using previous segmentation techniques. Based on this evaluation by clinicians, we concluded that there were no significant differences, indicating minimal errors in our automated segmentation process. For more accurate segmentation, only 3D thin-cut sagittal T1-weighted images, with a slice thickness of <1 mm from the initial scans, were utilized. Even with a relatively small sample size, our model showed stable predictive performance by treating multiple MRI scans of the same patient during hospitalization as separate cases for training. Before conducting MMDL, feature vectors were extracted from each modality and combined while minimizing information loss.

To capture subtle changes in the brain, we segmented the brain for analysis. However, since the brain is a continuous single structure, it is practically impossible for inflammation to affect only a single brain subregion, even when it is localized.^25^ To create a model that better reflects real clinical situations, we selected brain areas for use in MMDL through a grouping process. During multimodal deep learning, brain MRI data were processed through a CNN model to create feature vectors. Subsequently, these feature vectors underwent MLP modelling, which represents a form of joint fusion technique. Unlike simple late fusion methods that only combine prediction values, this approach, although computationally more intensive, enables more real-world applicable predictions.^23^

The brain MRI-based unimodal model outperformed the clinical variable-based model in predicting outcomes for viral infections. Unlike other causes, viral infections can invade brain parenchyma or be confined to the meninges, leading to distinct prognostic differences and clear radiological distinctions.^26,27^ Based on this, performing brain MRI scans when viral brain infection is suspected could be particularly helpful in prognostic prediction. In the case of autoimmune inflammation, the disease spectrum is broader and relatively heterogeneous compared to other infectious diseases.^28^ Hence, the final predictive performance is presumed to be the most unsatisfactory. This issue was also encountered in our previous clinical feature-based model for predicting the aetiology.^20^ To overcome this, recruiting a larger patient population for more detailed classification will be necessary in the future. Tuberculous infection also had a small number of patients included in the external test set. Therefore, we anticipate that recruiting more patients in the future will lead to a more stable predictive performance. Our model exhibited higher predictive performance compared to clinicians with varying levels of experience. All clinicians experienced a significant improvement in predictive accuracy, indicating the potential of AI-assisted care in CNS inflammatory diseases. These findings suggest that our model could be utilized as a decision-support tool during the initial assessment.

As recent studies highlight the importance of separate feature selection within each modality in multimodal integrations for enhanced accuracy and interpretability, and its systematic application for application and comprehension, a feature selection method was constructed and applied independently in clinical and MRI datasets.^29^ The clinical variables selected for MMDL differed depending on the aetiology. In patients with bacterial infection, inflammatory markers such as lactate and procalcitonin were prominently utilized.^30^ Overall, the level of consciousness at admission was crucially utilized, which is consistent with clinical prior knowledge.^31^ The brain regions identified as important in MRI also varied slightly depending on the cause. This is consistent with previous research indicating that the brain areas predominantly affected vary depending on the pathogen.^7^ Autoimmune and viral aetiologies are known to frequently involve the limbic system and temporal lobe.^26,28,32^ Consistently, in our study, these regions emerged as important predictors for prognosis. Upon examining the importance of variables used for prediction after MMDL, we observed a balanced distribution of clinical and brain imaging-related variables. In bacterial infections, CSF white blood cell count emerged as a strong prognostic factor compared to other variables.^33^ This underscores the importance of paying close attention to CSF test results in clinical practice. In patients with bacterial infections, changes in the occipital lobe, including the pericalcarine cortex, cuneus, and lingual gyrus, were identified as important variables for prognostic prediction. While brain abscesses are commonly associated with frontal and temporal lobes in bacterial infections, they can also occur in the occipital lobe, facilitated by the lateral ventricle.^34^ Although previous studies reported no difference in prognosis based on the location of brain abscesses,^35^ our findings suggest that changes in the occipital lobe should also be considered significant. In viral infections, brain subregions within the temporal lobe were identified as important for prognostic prediction in the multimodal model. Given that herpes viruses, which invade the temporal lobe, account for a considerable portion of viral brain infections, these results are considered reasonable.^36^ The high SHAP value in autoimmune cases was not confined to specific brain regions. This may be due to the heterogeneity of autoimmune encephalitis, as different regions may be affected depending on the antibodies involved. Meanwhile, serum creatine kinase levels, which can increase with frequent seizures, status epilepticus, or severe agitation, showed high SHAP values, indicating their influence on outcomes in autoimmune inflammation.^37^ After MMDL, clustering based on SHAP values revealed a tendency for patient clusters to group according to mRS scores in the UMAP plot. Particularly, we observed distinct clusters for patients with very poor outcomes (mRS 5,6) and those with less severe outcomes (mRS 3,4). Based on this, we can expand from binary classification model to multiclassification in the future with additional data to consider for more realistic predictive modelling.

### Limitation

The present study has some limitations. First, due to the small sample size, patients with fungal and parasitic infections could not be included in the study, and the number of patients with bacterial and tuberculosis infections was relatively low in the external validation dataset. Second, since we divided the CNS inflammation into four broad categories, we were unable to predict specific pathogens such as auto-antibodies or species of bacteria at the individual level. Third, there may be differences when applying the results of this study to patients of other races as most of the population included in this study are of Asian ethnicity. Finally, due to a lack of a sufficient number of thin-cut images necessary for training, we were unable to utilize T2-weighted brain MRI imaging, widely used in encephalitis patients. The previously mentioned limitations, including the small sample size and the inability to utilize all MRI protocols, may reduce the generalizability of this study. Therefore, we believe that future collection of brain imaging data from a larger cohort of patients could enhance the proposed MMDL model.

## Conclusion

In this study, we developed a successful MMDL model using brain MRI and clinical data to predict prognosis of CNS inflammation. This model not only outperformed clinical experts but also helped their decision-making process. A possible extension of this work can be automated measurement of changes in cortical thickness, which is a challenging and important process.

## Supplementary Material

fcaf179_Supplementary_Data

## Data Availability

The complete source code is made available on our GitHub repository. (https://github.com/DigitalHealthcareLab/24CNSInfectionMRI).
