# Supplementary material for: Multimodal deep learning model for prediction of prognosis in central nervous system inflammation
Source: Brain Commun. 2025 May 9;7(3):fcaf179. doi: 10.1093/braincomms/fcaf179 (PMC12082089; doi:10.1093/braincomms/fcaf179)
Supplement: fcaf179_Supplementary_Data [file fcaf179_supplementary_data.docx]

Appendix1. Pseudocode for approximated SHAP calculation method

**Algorithm 1** Approximating the contribution *ϕi* of the *i*th feature vector *⃗x_i_* out of a total of *k* feature vectors given instance *⃗x* = [*⃗x*_1_*, ⃗x*_2_*, . . . , ⃗x_k_*] *∈ X*, for model *f* , by drawing *m* samples.

*ϕ*ˆ*i ←* 0;

**for** 1 to *m* **do**

select, at random, *⃗z ∈ X*;

select, at random, coalition *ω⃗* = [*ω*_1_*, ω*_2_*, . . . , ω_k_*] *∈* ℤ*^k^*_2_;

construct new instance *⃗s* = [*⃗s*_1_*, ⃗s*_2_*, . . . , ⃗s_k_*];

**for** each *ω_j_ ∈ ω⃗ , j ≠* *i* **do**

**if** *ω_j_* == 1 **then**

*⃗s_j_ ← ⃗x_j_*;

else

*⃗s_j_ ← ⃗z_j_*;

end if end for

construct two new instances *⃗s*+ *← ⃗s*, *⃗s− ← ⃗s*;

*⃗s_i_*+ *← ⃗x_i_*;

*⃗s_i_− ← ⃗z_i_*;

*ϕ*ˆ*i ← ϕ*ˆ*i* + *f* (*⃗s*+ ) – *f* (*⃗s−* );

end for

*ϕ*ˆ*i ← ϕ*ˆ*i* / *m*

**Appendix 2. Hyperparameters of each model**

Unimodal CNN models

patience = 10, eval_start_epoch = 40, min_delta = 5e-4, learning_rate: 5e-5, batch_size = 4, weight decay = 0.025

Unimodal & Multimodal MLP models (by etiology)

Autoimmune – patience: 100, eval_start_epoch: 30, min_delta: 5e-5, learning_rate: 5e-7, batch_size: 32, weight decay = 0.1, T_0: 30 (CosineAnnealingWarmRestarts scheduler)

Bacterial – patience: 100, eval_start_epoch: 30, min_delta: 5e-5, learning_rate: 1e-7, batch_size: 32, weight decay = 0.1, T_0: 30 (CosineAnnealingWarmRestarts scheduler)

Tuberculosis – patience: 100, eval_start_epoch: 30, min_delta: 5e-5, learning_rate: 1e-7, batch_size: 32, weight decay = 0.1, T_0: 30 (CosineAnnealingWarmRestarts scheduler)

Viral – patience: 100, eval_start_epoch: 30, min_delta: 5e-5, learning_rate: 5e-7, batch_size: 32, weight decay = 0.1, T_0: 30 (CosineAnnealingWarmRestarts scheduler)

Feature Importance Calculation

num_to_sample: 6000

UMAP

n_components: 2, n_neighbors: 5, min_dist: 0.5, metric: 'correlation'

DBSCAN

eps: 1, min_samples: 3

**Supplementary Table 1. Feature distribution for each aetiology of central nervous system inflammation patients at Severance hospital**

|  | | | Internal dataset (2010.01.01 – 2020.12.31) | | | | | | | | | | External dataset (2021.01.01 – 2023.12.31) | | | | | | | | |  |
| --- | --- | --- | --- | --- | --- | --- | --- | --- | --- | --- | --- | --- | --- | --- | --- | --- | --- | --- | --- | --- | --- | --- |
| Aetiologies | | | Autoimmune  (n = 45) | | Bacterial  (n = 46) | | Tuberculosis  (n = 30) | | Viral  (n = 170) | | Overall  (n = 291) | | Autoimmune  (n = 29) | | Bacterial  (n = 8) | | Tuberculosis  (n = 3) | | Viral  (n = 66) | | Overall  (n = 106) |  |
| Total number of brain MRI images | | | 73 | | 75 | | 43 | | 222 | | 413 | | 78 | | 15 | | 5 | | 113 | | 211 |  |
| Age | | | 44.6 ± 19.0 | | 58.2 ± 18.5 | | 53.5 ± 18.6 | | 45.5 ± 19.3 | | 45.5 ± 19.3 | | 49.3 ± 19.0 | | 61.5 ± 19.8 | | 49.0 ± 19.0 | | 45.5 ± 18.9 | | 45.5 ± 18.9 |  |
| Female sex | | | 24 (53.3%) | | 20 (43.5%) | | 16 (53.3%) | | 80 (47.1%) | | 140 (48.1%) | | 14 (48.3%) | | 3 (37.5%) | | 0 (0%) | | 30 (45.5%) | | 47 (44.3%) |  |
| Poor prognosis (mRS > 2) | | | 14 (31.1%) | | 8 (17.4%) | | 7 (23.3%) | | 25 (14.7%) | | 54 (18.6%) | | 15 (51.7%) | | 2 (25%) | | 1 (33.3%) | | 13 (19.7%) | | 31 (29.2%) |  |
| Length of stay (day) | | | 53.6 ± 105.3 | | 48.7 ± 56.2 | | 48.3 ± 48.9 | | 18.4 ± 35.6 | | 31.8 ± 59.1 | | 26.9 ± 22.0 | | 26.9 ± 19.3 | | 18.3 ± 1.5 | | 16.2 ± 21.6 | | 19.8 ± 21.5 |  |
| Body mass index | | | 22.5 ± 2.7 | | 22.9 ± 3.2 | | 21.6 ± 1.9 | | 22.7 ± 2.2 | | 22.7 ± 2.2 | | 23.4 ± 2.7 | | 23.5 ± 3.7 | | 23.2 ± 3.5 | | 23.3 ± 3.7 | | 23.3 ± 3.7 |  |
| Comorbidities | | |  | |  | |  | |  | |  | |  | |  | |  | |  | |  |  |
| Charlson comorbidity index | | | 1.2 ± 1.8 | | 1.5 ± 1.9 | | 1.2 ± 1.8 | | 0.8 ± 1.5 | | 0.8 ± 1.5 | | 0.7 ± 1.0 | | 2.6 ± 1.5 | | 0.3 ± 0.6 | | 0.7 ± 1.4 | | 0.7 ± 1.4 |  |
| Seizure | | | 15 (33.3%) | | 10 (21.7%) | | 6 (20.0%) | | 24 (14.1%) | | 55 (18.9%) | | 10 (34.5%) | | 0 (0%) | | 0 (0%) | | 2 (3.0%) | | 12 (11.3%) |  |
| Abnormal mentality at admission | | | 19 (42.2%) | | 22 (47.8%) | | 8 (26.7%) | | 43 (25.3%) | | 92 (31.6%) | | 16 (55.2%) | | 6 (75.0%) | | 3 (100.0%) | | 16 (24.2%) | | 41 (38.7%) |  |
| Vital signs | | |  | |  | |  | |  | |  | |  | |  | |  | |  | |  |  |
| Mean systolic blood pressure (mmHg) | | | 127.9 ± 16.0 | | 131.5 ± 17.2 | | 133.4 ± 19.0 | | 128.4 ± 15.9 | | 128.4 ± 15.9 | | 128.2 ± 11.7 | | 125.8 ± 24.7 | | 124.5 ± 18.7 | | 126.3 ± 17.3 | | 126.3 ± 17.3 |  |
| Mean diastolic blood pressure (mmHg) | | | 74.8 ± 9.2 | | 73.7 ± 9.0 | | 79.4 ± 11.6 | | 76.6 ± 10.0 | | 76.6 ± 10.0 | | 78.9 ± 7.7 | | 70.1 ± 12.8 | | 74.8 ± 21.7 | | 78.6 ± 10.5 | | 78.6 ± 10.5 |  |
| Mean heart rate (rate/min) | | | 84.0 ± 13.7 | | 86.9 ± 15.8 | | 83.9 ± 11.7 | | 79.0 ± 11.9 | | 79.0 ± 11.9 | | 81.2 ± 11.9 | | 91.8 ± 17.8 | | 72.5 ± 18.7 | | 78.7 ± 12.5 | | 78.7 ± 12.5 |  |
| Mean respiratory rate (rate/min) | | | 18.9 ± 2.0 | | 18.8 ± 2.1 | | 18.5 ± 1.1 | | 18.6 ± 1.7 | | 18.6 ± 1.7 | | 18.5 ± 1.3 | | 21.8 ± 3.1 | | 18.6 ± 1.1 | | 18.8 ± 0.9 | | 18.8 ± 0.9 |  |
| Maximum body temperature (°C) | | | 37.2 ± 0.4 | | 37.4 ± 0.5 | | 37.4 ± 0.6 | | 37.4 ± 0.5 | | 37.4 ± 0.5 | | 37.6 ± 0.7 | | 38.5 ± 0.6 | | 37.7 ± 0.5 | | 38.1 ± 0.9 | | 38.1 ± 0.9 |  |
| Mean body temperature (°C) | | | 38.0 ± 0.8 | | 38.2 ± 0.7 | | 38.1 ± 0.6 | | 38.0 ± 0.7 | | 38.0 ± 0.7 | | 37.0 ± 0.5 | | 37.3 ± 0.5 | | 37.0 ± 0.3 | | 37.2 ± 0.5 | | 37.2 ± 0.5 |  |
| CSF laboratory findings |  |  | |  | |  | |  | |  | |  | |  | |  | |  | |  | | |
| WBC count (/μL) | | | 92.9 ± 158.3 | | 1143.8 ± 2016.2 | | 241.8 ± 333.9 | | 165.3 ± 238.1 | | 165.3 ± 238.1 | | 72.1 ± 121.2 | | 1059.8 ± 1241.2 | | 377.7 ± 464.7 | | 117.2 ± 108.4 | | 117.2 ± 108.4 |  |
| Mononuclear leukocyte ratio | | | 42.7 ± 42.8 | | 25.0 ± 30.7 | | 76.9 ± 27.9 | | 73.3 ± 33.9 | | 73.3 ± 33.9 | | 63.0 ± 42.0 | | 24.4 ± 32.3 | | 62.7 ± 47.4 | | 73.4 ± 23.2 | | 73.4 ± 23.2 |  |
| Polymorphonuclear leukocyte ratio | | | 11.1 ± 22.5 | | 61.9 ± 34.4 | | 16.7 ± 24.6 | | 8.6 ± 20.4 | | 8.6 ± 20.4 | | 10.3 ± 24.6 | | 56.6 ± 33.5 | | 27.0 ± 45.9 | | 11.4 ± 19.8 | | 11.4 ± 19.8 |  |
| Basophil ratio | | | 0.2 ± 0.6 | | 0.1 ± 0.4 | | 0.1 ± 0.3 | | 0.4 ± 1.0 | | 0.4 ± 1.0 | | 0.2 ± 0.6 | | 0.0 ± 0.0 | | 0.3 ± 0.6 | | 0.2 ± 0.5 | | 0.2 ± 0.5 |  |
| Eosinophil ratio | | | 0.0 ± 0.3 | | 0.1 ± 0.2 | | 0.1 ± 0.3 | | 0.2 ± 0.7 | | 0.2 ± 0.7 | | 0.1 ± 0.4 | | 0.1 ± 0.4 | | 0.0 ± 0.0 | | 0.1 ± 0.7 | | 0.1 ± 0.7 |  |
| Protein (mg/dL) | | | 71.3 ± 53.4 | | 319.4 ± 344.2 | | 584.3 ± 1345.7 | | 115.0 ± 205.8 | | 115.0 ± 205.8 | | 77.9 ± 69.7 | | 287.5 ± 270.3 | | 227.6 ± 97.7 | | 121.3 ± 118.3 | | 121.3 ± 118.3 |  |
| Glucose (mg/dL) | | | 71.2 ± 23.4 | | 42.7 ± 27.1 | | 47.6 ± 33.6 | | 63.7 ± 17.9 | | 63.7 ± 17.9 | | 69.1 ± 22.7 | | 50.4 ± 20.3 | | 57.3 ± 18.0 | | 59.1 ± 11.7 | | 59.1 ± 11.7 |  |
| CSF/serum glucose ratio | | | 0.6 ± 0.1 | | 0.3 ± 0.2 | | 0.3 ± 0.2 | | 0.5 ± 0.1 | | 0.5 ± 0.1 | | 0.6 ± 0.2 | | 0.4 ± 0.2 | | 0.4 ± 0.1 | | 0.5 ± 0.1 | | 0.5 ± 0.1 |  |
| RBC count > 100/μL | | | 6 (13.3%) | | 16 (34.8%) | | 4 (13.3%) | | 26 (15.3%) | | 52 (17.9%) | | 1 (3.4%) | | 2 (25.0%) | | 1 (33.3%) | | 11 (16.7%) | | 15 (14.2%) |  |
| Specific gravity | | | 1.0 ± 0.0 | | 1.0 ± 0.0 | | 1.0 ± 0.0 | | 1.0 ± 0.0 | | 1.0 ± 0.0 | | 1.0 ± 0.0 | | 1.0 ± 0.0 | | 1.0 ± 0.0 | | 1.0 ± 0.0 | | 1.0 ± 0.0 |  |
| Turbidity | | | 4 (8.9%) | | 24 (52.2%) | | 9 (30.0%) | | 21 (12.4%) | | 58 (19.9%) | | 2 (6.9%) | | 4 (50.0%) | | 0 (0%) | | 10 (15.2%) | | 16 (15.1%) |  |
| Adenosine deaminase (IU/L) | | | 2.9 ± 2.0 | | 6.2 ± 5.3 | | 21.0 ± 40.0 | | 4.5 ± 3.2 | | 4.5 ± 3.2 | | 3.2 ± 3.4 | | 6.0 ± 4.6 | | 16.4 ± 9.3 | | 3.7 ± 2.7 | | 3.7 ± 2.7 |  |
| Abnormal colour | | | 4 (8.9%) | | 20 (43.5%) | | 14 (46.7%) | | 15 (8.8%) | | 53 (18.2%) | | 1 (3.4%) | | 4 (50.0%) | | 0 (0%) | | 7 (10.6%) | | 12 (11.3%) |  |
| High pH | | | 30 (66.7%) | | 26 (56.5%) | | 17 (56.7%) | | 134 (78.8%) | | 207 (71.1%) | | 22 (75.9%) | | 5 (62.5%) | | 2 (66.7%) | | 63 (95.5%) | | 92 (86.8%) |  |
| Blood laboratory findings |  |  | |  | |  | |  | |  | |  | |  | |  | |  | |  | | |
| WBC count (10^9^/L) | | | 9.3 ± 5.0 | | 12.6 ± 7.4 | | 7.9 ± 4.4 | | 8.5 ± 4.0 | | 8.5 ± 4.0 | | 8.2 ± 2.5 | | 14.3 ± 4.2 | | 5.4 ± 3.3 | | 8.0 ± 3.0 | | 8.0 ± 3.0 |  |
| Neutrophil ratio | | | 75.2 ± 13.5 | | 81.8 ± 12.1 | | 74.0 ± 11.6 | | 72.0 ± 11.3 | | 72.0 ± 11.3 | | 69.5 ± 12.6 | | 80.2 ± 9.0 | | 60.1 ± 20.2 | | 71.2 ± 11.9 | | 71.2 ± 11.9 |  |
| Monocyte ratio | | | 5.0 ± 2.2 | | 4.5 ± 2.1 | | 6.4 ± 2.4 | | 5.5 ± 1.9 | | 5.5 ± 1.9 | | 7.1 ± 3.3 | | 5.2 ± 3.3 | | 8.3 ± 2.5 | | 6.3 ± 3.2 | | 6.3 ± 3.2 |  |
| Basophil ratio | | | 0.4 ± 0.4 | | 0.2 ± 0.2 | | 0.3 ± 0.2 | | 0.4 ± 0.3 | | 0.4 ± 0.3 | | 0.3 ± 0.2 | | 0.3 ± 0.1 | | 0.2 ± 0.2 | | 0.4 ± 0.3 | | 0.4 ± 0.3 |  |
| Eosinophil ratio | | | 1.2 ± 1.3 | | 0.8 ± 0.7 | | 1.5 ± 1.9 | | 1.3 ± 1.1 | | 1.3 ± 1.1 | | 1.3 ± 1.4 | | 1.1 ± 1.1 | | 2.4 ± 3.5 | | 1.4 ± 1.3 | | 1.4 ± 1.3 |  |
| Large unstained cells ratio | | | 1.5 ± 0.8 | | 1.5 ± 1.0 | | 1.7 ± 0.8 | | 2.1 ± 1.4 | | 2.1 ± 1.4 | | 2.1 ± 0.9 | | 1.2 ± 0.9 | | 2.0 ± 0.6 | | 1.8 ± 1.1 | | 1.8 ± 1.1 |  |
| Haemoglobin (g/dL) | | | 13.4 ± 1.8 | | 12.4 ± 2.2 | | 13.0 ± 2.1 | | 13.5 ± 1.5 | | 13.5 ± 1.5 | | 13.5 ± 1.8 | | 13.3 ± 1.8 | | 14.1 ± 0.9 | | 13.7 ± 1.6 | | 13.7 ± 1.6 |  |
| Platelet count (10^9^/L) | | | 242.6 ± 92.5 | | 218.0 ± 106.1 | | 264.0 ± 115.6 | | 242.2 ± 76.5 | | 242.2 ± 76.5 | | 265.3 ± 81.1 | | 242.5 ± 141.0 | | 222.0 ± 76.4 | | 241.3 ± 73.5 | | 241.3 ± 73.5 |  |
| Mean platelet volume (fL) | | | 7.8 ± 1.0 | | 8.3 ± 1.1 | | 7.6 ± 0.6 | | 7.9 ± 0.9 | | 7.9 ± 0.9 | | 8.7 ± 1.2 | | 9.0 ± 2.6 | | 9.2 ± 2.4 | | 8.7 ± 1.3 | | 8.7 ± 1.3 |  |
| Red cell distribution width (%) | | | 13.1 ± 1.1 | | 13.8 ± 2.1 | | 14.0 ± 1.8 | | 13.0 ± 1.4 | | 13.0 ± 1.4 | | 13.0 ± 1.1 | | 14.9 ± 1.9 | | 12.7 ± 1.3 | | 13.2 ± 1.4 | | 13.2 ± 1.4 |  |
| Prothrombin time (INR) | | | 1.1 ± 0.4 | | 1.1 ± 0.1 | | 1.0 ± 0.1 | | 1.0 ± 0.1 | | 1.0 ± 0.1 | | 1.0 ± 0.1 | | 1.1 ± 0.1 | | 0.9 ± 0.0 | | 1.0 ± 0.3 | | 1.0 ± 0.3 |  |
| aPTT (sec) | | | 29.9 ± 4.3 | | 29.6 ± 4.6 | | 28.8 ± 3.6 | | 30.0 ± 3.5 | | 30.0 ± 3.5 | | 28.9 ± 3.5 | | 32.1 ± 8.2 | | 31.5 ± 1.0 | | 32.3 ± 4.3 | | 32.3 ± 4.3 |  |
| Thrombotic microangiopathy > 2+ | | | 12 (63.2%) | | 10 (52.6%) | | 8 (47.1%) | | 50 (48.1%) | | 80 (50.3%) | | 10 (34.5%) | | 5 (62.5%) | | 1 (33.3%) | | 27 (40.9%) | | 43 (40.6%) |  |
| Sodium ion concentration (mmol/L) | | | 137.2 ± 4.8 | | 136.4 ± 4.7 | | 134.1 ± 4.5 | | 137.5 ± 3.9 | | 137.5 ± 3.9 | | 136.7 ± 5.4 | | 133.5 ± 6.0 | | 138.0 ± 2.6 | | 136.6 ± 4.4 | | 136.6 ± 4.4 |  |
| Potassium ion concentration (mmol/L) | | | 4.1 ± 0.5 | | 4.1 ± 0.5 | | 4.0 ± 0.5 | | 4.1 ± 0.4 | | 4.1 ± 0.4 | | 4.2 ± 0.6 | | 4.5 ± 0.9 | | 3.8 ± 0.3 | | 4.1 ± 0.4 | | 4.1 ± 0.4 |  |
| tCO2 (mmol/L) | | | 22.2 ± 3.6 | | 22.3 ± 3.0 | | 22.9 ± 3.3 | | 22.9 ± 3.0 | | 22.9 ± 3.0 | | 24.8 ± 2.7 | | 21.2 ± 3.0 | | 26.0 ± 0.0 | | 24.6 ± 2.5 | | 24.6 ± 2.5 |  |
| Blood urea nitrogen (mg/dL) | | | 14.6 ± 8.0 | | 18.8 ± 12.0 | | 14.6 ± 12.3 | | 14.5 ± 7.9 | | 14.5 ± 7.9 | | 17.1 ± 5.7 | | 17.1 ± 5.8 | | 17.1 ± 5.9 | | 17.1 ± 5.10 | | 15.3 ± 5.9 |  |
| Creatinine (mg/dL) | | | 0.9 ± 1.2 | | 1.3 ± 1.9 | | 0.7 ± 0.5 | | 0.8 ± 0.3 | | 0.8 ± 0.3 | | 0.8 ± 0.3 | | 1.3 ± 0.8 | | 0.7 ± 0.1 | | 0.8 ± 0.2 | | 0.8 ± 0.2 |  |
| Glucose (mg/dL) | | | 128.0 ± 41.8 | | 147.7 ± 47.4 | | 131.7 ± 54.3 | | 123.4 ± 34.0 | | 123.4 ± 34.0 | | 119.7 ± 41.7 | | 133.6 ± 41.5 | | 122.0 ± 27.4 | | 118.2 ± 35.0 | | 118.2 ± 35.0 |  |
| Albumin (g/dL) | | | 4.1 ± 0.5 | | 3.7 ± 0.7 | | 3.8 ± 0.6 | | 4.2 ± 0.5 | | 4.2 ± 0.5 | | 4.2 ± 0.6 | | 4.0 ± 0.5 | | 4.0 ± 0.5 | | 4.4 ± 0.4 | | 4.4 ± 0.4 |  |
| Aspartate transaminase (IU/L) | | | 42.3 ± 48.2 | | 31.1 ± 20.7 | | 27.4 ± 32.6 | | 28.1 ± 21.3 | | 28.1 ± 21.3 | | 26.8 ± 13.0 | | 61.0 ± 59.8 | | 27.0 ± 9.2 | | 32.6 ± 42.4 | | 32.6 ± 42.4 |  |
| Alanine aminotransferase (IU/L) | | | 37.5 ± 39.9 | | 27.9 ± 24.0 | | 19.4 ± 13.1 | | 26.4 ± 28.5 | | 26.4 ± 28.5 | | 38.7 ± 37.8 | | 39.8 ± 29.7 | | 27.3 ± 10.7 | | 32.4 ± 48.1 | | 32.4 ± 48.1 |  |
| Total bilirubin (mg/dL) | | | 0.8 ± 0.7 | | 0.7 ± 0.5 | | 0.9 ± 0.8 | | 0.7 ± 0.3 | | 0.7 ± 0.3 | | 0.8 ± 0.4 | | 0.9 ± 0.6 | | 0.7 ± 0.2 | | 0.8 ± 0.4 | | 0.8 ± 0.4 |  |
| Alkaline phosphatase (IU/L) | | | 66.5 ± 33.2 | | 85.2 ± 37.8 | | 72.0 ± 52.1 | | 66.3 ± 33.0 | | 66.3 ± 33.0 | | 67.9 ± 20.1 | | 93.5 ± 33.4 | | 76.3 ± 20.0 | | 68.6 ± 29.7 | | 68.6 ± 29.7 |  |
| Uric acid (mg/dL) | | | 4.4 ± 1.4 | | 4.0 ± 1.5 | | 4.4 ± 2.8 | | 4.0 ± 1.5 | | 4.0 ± 1.5 | | 4.7 ± 1.7 | | 6.6 ± 3.9 | | 3.0 ± 0.4 | | 4.0 ± 1.4 | | 4.0 ± 1.4 |  |
| Inorganic phosphorus (mg/dL) | | | 3.2 ± 0.8 | | 3.0 ± 0.8 | | 3.3 ± 0.7 | | 3.3 ± 0.7 | | 3.3 ± 0.7 | | 3.6 ± 0.7 | | 3.5 ± 1.2 | | 3.3 ± 1.1 | | 3.3 ± 0.8 | | 3.3 ± 0.8 |  |
| Calcium (mg/dL) | | | 8.8 ± 0.6 | | 8.8 ± 0.7 | | 8.7 ± 0.6 | | 8.9 ± 0.5 | | 8.9 ± 0.5 | | 8.9 ± 0.5 | | 8.6 ± 0.5 | | 8.7 ± 0.5 | | 8.9 ± 0.5 | | 8.9 ± 0.5 |  |
| Creatinine Kinase (IU/L) | | | 602.2 ± 1491.2 | | 204.2 ± 345.7 | | 135.5 ± 201.9 | | 419.8 ± 757.3 | | 419.8 ± 757.3 | | 221.9 ± 547.9 | | 953.2 ± 1455.3 | | - | | 120.4 ± 114.7 | | 120.4 ± 114.7 |  |
| Ammonia (μg/dL) | | | 51.1 ± 29.5 | | 44.9 ± 29.0 | | 51.9 ± 19.5 | | 45.0 ± 16.5 | | 45.0 ± 16.5 | | 36.1 ± 12.4 | | 30.2 ± 7.1 | | 41.5 ± 27.6 | | 44.8 ± 37.7 | | 44.8 ± 37.7 |  |
| Total cholesterol (mg/dL) | | | 157.1 ± 32.1 | | 153.3 ± 48.5 | | 174.7 ± 38.8 | | 163.9 ± 34.9 | | 163.9 ± 34.9 | | 173.7 ± 47.8 | | 153.2 ± 62.5 | | 155.0 ± 0.0 | | 163.0 ± 45.2 | | 163.0 ± 45.2 |  |
| C-reactive protein (mg/L) | | | 29.5 ± 51.1 | | 87.6 ± 82.8 | | 24.2 ± 49.0 | | 22.5 ± 40.5 | | 22.5 ± 40.5 | | 11.0 ± 19.8 | | 111.1 ± 94.9 | | 23.3 ± 33.4 | | 20.0 ± 37.2 | | 20.0 ± 37.2 |  |
| Erythrocyte sedimentation rate (mm/hr) | | | 27.0 ± 30.2 | | 52.6 ± 34.4 | | 43.4 ± 39.1 | | 28.7 ± 26.0 | | 28.7 ± 26.0 | | 18.4 ± 18.6 | | 58.9 ± 37.0 | | 8.5 ± 6.4 | | 18.4 ± 23.8 | | 18.4 ± 23.8 |  |
| Procalcitonin (ng/mL) | | | 0.4 ± 0.9 | | 8.2 ± 20.1 | | 0.1 ± 0.1 | | 0.1 ± 0.2 | | 0.1 ± 0.2 | | 0.1 ± 0.2 | | 1.8 ± 2.6 | | 0.1 ± 0.1 | | 0.1 ± 0.1 | | 0.1 ± 0.1 |  |
| Lactate (mmol/L) | | | 1.7 ± 1.4 | | 2.5 ± 1.9 | | 1.0 ± 0.3 | | 1.3 ± 0.8 | | 1.3 ± 0.8 | | 1.0 ± 0.4 | | 1.2 ± 0.7 | | - | | 2.7 ± 3.4 | | 2.7 ± 3.4 |  |
| Urine laboratory findings |  |  | |  | |  | |  | |  | |  | |  | |  | |  | |  | | |
| Bilirubinuria | | | 4 (9.5%) | | 6 (14.6%) | | 7 (24.1%) | | 9 (5.8%) | | 26 (9.8%) | | 2 (6.9%) | | 3 (37.5%) | | 0 (0%) | | 8 (12.1%) | | 13 (12.3%) |  |
| Haematuria | | | 14 (33.3%) | | 15 (36.6%) | | 12 (41.4%) | | 56 (36.4%) | | 97 (36.5%) | | 5 (17.2%) | | 1 (12.5%) | | 2 (66.7%) | | 13 (19.7%) | | 21 (19.8%) |  |
| Glucosuria | | | 6 (14.3%) | | 13 (31.7%) | | 5 (17.2%) | | 23 (14.9%) | | 47 (17.7%) | | 5 (17.2%) | | 5 (62.5%) | | 1 (33.3%) | | 4 (6.1%) | | 15 (14.2%) |  |
| Ketonuria | | | 20 (47.6%) | | 26 (63.4%) | | 14 (48.3%) | | 76 (49.4%) | | 136 (51.1%) | | 6 (20.7%) | | 3 (37.5%) | | 1 (33.3%) | | 24 (36.4%) | | 34 (32.1%) |  |
| High leukocyte esterase | | | 10 (23.8%) | | 9 (22.0%) | | 13 (44.8%) | | 39 (25.3%) | | 71 (26.7%) | | 4 (13.8%) | | 1 (12.5%) | | 1 (33.3%) | | 11 (16.7%) | | 17 (16.0%) |  |
| Albuminuria | | | 13 (31.0%) | | 28 (68.3%) | | 9 (31.0%) | | 53 (34.4%) | | 103 (38.7%) | | 2 (6.9%) | | 0 (0%) | | 0 (0%) | | 5 (7.6%) | | 7 (6.6%) |  |
| High RBC count | | | 11 (26.2%) | | 16 (39.0%) | | 12 (41.4%) | | 51 (33.1%) | | 90 (33.8%) | | 5 (17.2%) | | 2 (25.0%) | | 2 (66.7%) | | 11 (16.7%) | | 20 (18.9%) |  |
| High WBC count | | | 12 (28.6%) | | 11 (26.8%) | | 7 (24.1%) | | 49 (31.8%) | | 79 (29.7%) | | 7 (24.1%) | | 3 (37.5%) | | 1 (33.3%) | | 20 (30.3%) | | 31 (29.2%) |  |
| Treatment | | |  | |  | |  | |  | |  | |  | |  | |  | |  | |  |  |
| Steroid usage | | | 42 (93.3%) | | 37 (80.4%) | | 26 (86.7%) | | 91 (53.5%) | | 196 (67.4%) | | 28 (96.6%) | | 5 (62.5%) | | 3 (100%) | | 43 (65.2%) | | 79 (74.5%) |  |
| Immunotherapy | | | 29 (64.4%) | | 4 (8.7%) | | 2 (6.7%) | | 12 (7.1%) | | 47 (16.2%) | | 13 (44.8%) | | 1 (12.5%) | | 0 (0%) | | 3 (4.5%) | | 17 (16%) |  |
| Mechanical ventilator application | | | 15 (33.3%) | | 17 (37%) | | 9 (30%) | | 18 (10.6%) | | 59 (20.3%) | | 2 (6.9%) | | 1 (12.5%) | | 0 (0%) | | 1 (1.5%) | | 4 (3.8%) |  |
| Intensive care unit admission | | | 16 (35.6%) | | 18 (39.1%) | | 12 (40%) | | 23 (13.5%) | | 69 (23.7%) | | 2 (6.9%) | | 1 (12.5%) | | 0 (0%) | | 1 (1.5%) | | 4 (3.8%) |  |

**Supplementary Table 2. ICD-10 codes of meningitis and encephalitis used in this study**

| ICD-10 code | Diagnosis |
| --- | --- |
| A02.2 | Localized salmonella infections |
| A17.0 | Tuberculous meningitis |
| A17.1 | Meningeal tuberculoma |
| A17.8 | Other tuberculosis of nervous system |
| A17.81 | Tuberculoma of brain and spinal cord |
| A17.82 | Tuberculous meningoencephalitis |
| A17.88 | Other tuberculosis of nervous system |
| A17.9 | Tuberculosis of nervous system, unspecified |
| A20.3 | Plague meningitis |
| A32.1 | Listerial meningitis and meningoencephalitis |
| A39.0 | Meningococcal meningitis |
| A50.4 | Late congenital neurosyphilis [juvenile neurosyphilis] |
| A51.4 | Other secondary syphilis |
| A52.1 | Symptomatic neurosyphilis |
| A54.8 | Other gonococcal infections |
| A83 | Mosquito-borne viral encephalitis |
| A83.0 | Japanese encephalitis |
| A83.1 | Western equine encephalitis |
| A83.2 | Eastern equine encephalitis |
| A83.3 | St. Louis encephalitis |
| A83.4 | Australian encephalitis |
| A83.5 | California encephalitis |
| A83.8 | Other mosquito-borne viral encephalitis |
| A83.9 | Mosquito-borne viral encephalitis, unspecified |
| A84 | Tick-borne viral encephalitis |
| A84.0 | Far Eastern tick-borne encephalitis [Russian spring-summer encephalitis |
| A84.1 | Central European tick-borne encephalitis |
| A84.8 | Other tick-borne viral encephalitis |
| A84.9 | Tick-borne viral encephalitis, unspecified |
| A85 | Other viral encephalitis, NEC |
| A85.0 | Enteroviral encephalitis |
| A85.1 | Adenoviral encephalitis |
| A85.2 | Arthropod-borne viral encephalitis, unspecified |
| A85.8 | Other specified viral encephalitis |
| A86 | Unspecified viral encephalitis |
| A87 | Viral meningitis |
| A87.0 | Enteroviral meningitis |
| A87.1 | Adenoviral meningitis |
| A87.2 | Lymphocytic choriomeningitis |
| A87.8 | Other viral meningitis |
| A87.9 | Viral meningitis, unspecified |
| B00.3 | Herpesviral meningitis |
| B00.4 | Herpesviral encephalitis |
| B01.0 | Varicella meningitis |
| B01.1 | Varicella encephalitis |
| B02.0 | Zoster encephalitis |
| B02.1 | Zoster meningitis |
| B05.0 | Measles complicated by encephalitis |
| B05.1 | Measles complicated by meningitis |
| B06.0 | Rubella with neurological complications |
| B26.1 | Mumps meningitis |
| B26.2 | Mumps encephalitis |
| B37.5 | Candidal meningitis |
| B38.4 | Coccidioidomycosis meningitis |
| B58.2 | Toxoplasma meningoencephalitis |
| G00 | Bacterial meningitis, NEC |
| G00.0 | Haemophilus meningitis |
| G00.1 | Pneumococcal meningitis |
| G00.2 | Streptococcal meningitis |
| G00.3 | Staphylococcal meningitis |
| G00.8 | Other bacterial meningitis |
| G00.9 | Bacterial meningitis, unspecified |
| G01 | Meningitis in bacterial diseases classified elsewhere |
| G02 | Meningitis in other infectious and parasitic diseases classified elsewhere |
| G02.0 | Meningitis in viral diseases classified elsewhere |
| G02.1 | Meningitis in mycoses |
| G02.8 | Meningitis in other specified infectious and parasitic diseases classified elsewhere |
| G03 | Meningitis due to other and unspecified causes |
| G03.0 | Nonpyogenic meningitis |
| G03.1 | Chronic meningitis |
| G03.2 | Benign recurrent meningitis [Mollaret] |
| G03.8 | Meningitis due to other specified causes |
| G03.9 | Meningitis, unspecified |
| G04 | Encephalitis, myelitis and encephalomyelitis |
| G04.2 | Bacterial meningoencephalitis and meningomyelitis |
| G04.8 | Other encephalitis, myelitis and encephalomyelitis |
| G04.9 | Encephalitis, myelitis and encephalomyelitis, unspecified |
| G05 | Encephalitis, myelitis and encephalomyelitis in diseases classified elsewhere |
| G05.0 | Encephalitis, myelitis and encephalomyelitis in bacterial diseases classified elsewhere |
| G05.1 | Encephalitis, myelitis and encephalomyelitis in viral diseases classified elsewhere |
| G05.2 | Encephalitis, myelitis and encephalomyelitis in other infectious and parasitic diseases classified elsewhere |
| G05.8 | Encephalitis, myelitis and encephalomyelitis in other diseases classified elsewhere |
| G06 | Intracranial and intraspinal abscess and granuloma |
| G06.0 | Intracranial abscess and granuloma |
| G06.1 | Intraspinal abscess and granuloma |
| G06.2 | Extradural and subdural abscess, unspecified |
| G07 | Intracranial and intraspinal abscess and granuloma in diseases classified elsewhere |
| G36 | Other acute disseminated demyelination |

Supplementary Table 3. The prognostic performance according to segmented brain regions for each etiology from unimodal models using brain MRI.

| **Brainpart** | **Autoimmune** | **Bacteria** | **Tuberculosis** | **Virus** | **Overall** |
| --- | --- | --- | --- | --- | --- |
| Accumbens_area | 0.8661 | 0.9500 | 0.7917 | 0.7558 | 0.6990 |
| Amygdala | 0.7589 | 0.8750 | 0.6250 | 0.6542 | 0.7632 |
| Brain_Stem | 0.8214 | 1.0000 | 0.4444 | 0.9064 | 0.8196 |
| Caudate | 0.7054 | 1.0000 | 0.4583 | 0.5833 | 0.6799 |
| Cerebellum_Cortex | 0.4196 | 0.9750 | 0.8472 | 0.7727 | 0.7637 |
| Cerebellum_White_Matter | 0.4018 | 0.9750 | 0.8194 | 0.8202 | 0.7755 |
| Choroid_plexus | 0.6786 | 1.0000 | 0.1528 | 0.6192 | 0.6475 |
| Hippocampus | 0.7768 | 1.0000 | 0.7639 | 0.6462 | 0.5985 |
| Pallidum | 0.7411 | 0.9500 | 0.3889 | 0.6506 | 0.7527 |
| Putamen | 0.5089 | 0.8500 | 0.6389 | 0.8092 | 0.7404 |
| Thalamus_Proper | 0.7232 | 1.0000 | 0.5139 | 0.7858 | 0.7953 |
| VentralDC | 0.7946 | 1.0000 | 0.4028 | 0.7376 | 0.8358 |
| caudalanteriorcingulate | 0.8214 | 1.0000 | 0.4306 | 0.6367 | 0.6265 |
| caudalmiddlefrontal | 0.7054 | 0.9250 | 0.6806 | 0.6681 | 0.6752 |
| cuneus | 0.8304 | 0.7000 | 0.9444 | 0.5197 | 0.4782 |
| entorhinal | 0.6875 | 0.9500 | 0.6111 | 0.6681 | 0.7100 |
| fusiform | 0.6964 | 1.0000 | 0.8194 | 0.5804 | 0.6395 |
| inferiorparietal | 0.6786 | 0.9750 | 0.9583 | 0.6871 | 0.6456 |
| inferiortemporal | 0.9107 | 1.0000 | 0.9167 | 0.6206 | 0.6005 |
| insula | 0.7321 | 0.9750 | 0.3611 | 0.6411 | 0.6020 |
| isthmuscingulate | 0.7679 | 0.8500 | 0.6806 | 0.5051 | 0.5157 |
| lateraloccipital | 0.7768 | 0.8500 | 0.9722 | 0.6864 | 0.6054 |
| lateralorbitofrontal | 0.8482 | 1.0000 | 0.3472 | 0.6798 | 0.7975 |
| lingual | 0.5357 | 0.7750 | 0.4444 | 0.5914 | 0.5527 |
| medialorbitofrontal | 0.7589 | 1.0000 | 0.3611 | 0.7712 | 0.7517 |
| middletemporal | 0.6429 | 1.0000 | 0.8750 | 0.6250 | 0.6000 |
| paracentral | 0.5089 | 0.6000 | 0.6111 | 0.6923 | 0.5733 |
| parahippocampal | 0.8214 | 1.0000 | 0.4167 | 0.6806 | 0.6542 |
| parsopercularis | 0.8214 | 0.9000 | 0.4167 | 0.6696 | 0.6848 |
| parsorbitalis | 0.7232 | 0.9000 | 0.5972 | 0.8224 | 0.7064 |
| parstriangularis | 0.5893 | 0.9500 | 0.8333 | 0.6418 | 0.6870 |
| pericalcarine | 0.8214 | 0.6000 | 0.6389 | 0.3618 | 0.4850 |
| postcentral | 0.7321 | 1.0000 | 0.3611 | 0.5892 | 0.5902 |
| posteriorcingulate | 0.5714 | 0.7500 | 0.7500 | 0.6542 | 0.4598 |
| precentral | 0.6964 | 1.0000 | 0.5000 | 0.5322 | 0.6326 |
| precuneus | 0.7232 | 0.9500 | 0.8333 | 0.5906 | 0.6333 |
| rostralanteriorcingulate | 0.9821 | 0.9000 | 0.4444 | 0.5797 | 0.7088 |
| rostralmiddlefrontal | 0.5893 | 1.0000 | 0.4583 | 0.5658 | 0.6081 |
| superiorfrontal | 0.4643 | 1.0000 | 0.9861 | 0.5936 | 0.6745 |
| superiorparietal | 0.6786 | 1.0000 | 0.9028 | 0.6944 | 0.6417 |
| superiortemporal | 0.5446 | 1.0000 | 0.5278 | 0.6199 | 0.7010 |
| supramarginal | 0.7411 | 1.0000 | 0.8194 | 0.6352 | 0.6284 |
| transversetemporal | 0.4732 | 0.8250 | 0.5139 | 0.6535 | 0.6434 |

Supplementary Table 4. The Performance of prognostic prediction without providing information about the etiology in patients with central nervous system inflammation.

|  |  | AUROC | AUPRC | Accuracy | F1 score |
| --- | --- | --- | --- | --- | --- |
| Unimodal model with MRI data | Internal | 0.8715 | 0.5822 | 0.9157 | 0.7742 |
|  | External | 0.6690 | 0.5086 | 0.6967 | 0.6145 |
| Unimodal model with clinical dada | Internal | 0.7922 | 0.4424 | 0.6265 | 0.4918 |
|  | External | 0.6781 | 0.5256 | 0.6967 | 0.5616 |
| Multimodal model with MRI and clinical data | Internal | 0.8078 | 0.4837 | 0.7108 | 0.5200 |
|  | External | 0.7573 | 0.5541 | 0.7678 | 0.6573 |

* Abbreviations: AUROC, the area under the receiver operating characteristic curve; AUPRC, the area under the precision-recall curve.

Supplementary Table 5. Mean Shapley additive explanations (SHAP) values measured according to the aetiology within 14 groups clustered based on anatomical similarity. The top 10 groups selected for multimodal deep learning for each cause were marked with an asterisk (*) after the SHAP value.

| **Brain regions (groups)** | | **Etiologies** | | | |
| --- | --- | --- | --- | --- | --- |
|  |  | **Autoimmune** | **Bacteria** | **Tuberculosis** | **Virus** |
| Frontal  lobe | caudal middle frontal gyrus,  lateral orbitofrontal gyrus,  medial orbitofrontal gyrus, pars orbitalis,  pars opercularis, pars triangularis,  precentral gyrus, superior frontal gyrus,  rostral middle frontal gyrus | 1.97* | 1.87 | 0.30* | 0.36 |
| Medial temporal lobe | hippocampus, parahippocampal gyrus,  ethornial cortex | 8.23* | 1.47 | 0.48* | 0.38* |
| Superolateral / Inferior  temporal lobe | fusiform gyrus, superior temporal gyrus,  inferior temporal gyrus, middle temporal gyrus, transverse temporal gyrus | 2.76* | 3.89* | 0.26 | 0.51* |
| Cortical limbic system | caudal anterior cingulate,  rostral anterior cingulate,  isthmus of cingulate gyrus, posterior cingulate | 2.94* | 2.91* | 0.66* | 0.29 |
| Parietal lobe | paracentral lobule, postcentral gyrus, inferiorparietal lobule, precuneus,  superior parietal lobule, supramarginal gyrus | 2.32* | 2.22* | 0.62* | 0.37 |
| Occipital lobe | cuneus cortex, lateral occipital cortex,  lingual gyrus, pericalcarine cortex | 3.68* | 5.89* | 0.49* | 0.38* |
| Insular Cortex | | 1.02 | 5.58* | 0.57* | 0.41* |
| Basal Ganglia | nucleus accumbens, caudate nucleus,  putamen, globus pallidum | 3.53* | 2.92* | 0.53* | 0.31 |
| Thalamus | | 4.19* | 2.43* | 0.27* | 0.41* |
| Ventral diencephalon | | 8.98* | 2.64* | 0.16 | 0.68* |
| Amygdala | | 0.93 | 1.99 | 0.37* | 0.47* |
| Choroid plexus | | 5.31* | 2.11 | 0.51* | 0.75* |
| Cerebellum Areas | cerebellum cortex, cerebellum white matter | 1.08 | 2.79* | 0.21 | 0.78* |
| Brain Stem | | 0.43 | 2.64* | 0.26 | 0.63* |

Supplementary Figure 1. Flowchart of patient selection.

* Abbreviation: CSF, cerebrospinal fluid; MRI, magnetic resonance imaging

Supplementary Figure 2. The schematic diagram of the preprocessing pipeline

* Abbreviations: DICOM, Digital Imaging and Communications in Medicine; NIFTI, Neuroimaging Informatics Technology Initiative; LIA, left, inferior, and anterior; MGH, Massachusetts General Hospital

Supplementary Figure 3. Diagram illustrating the training strategy of the unimodal model based on brain MRI variables. Each fold was trained with a similar age distribution.


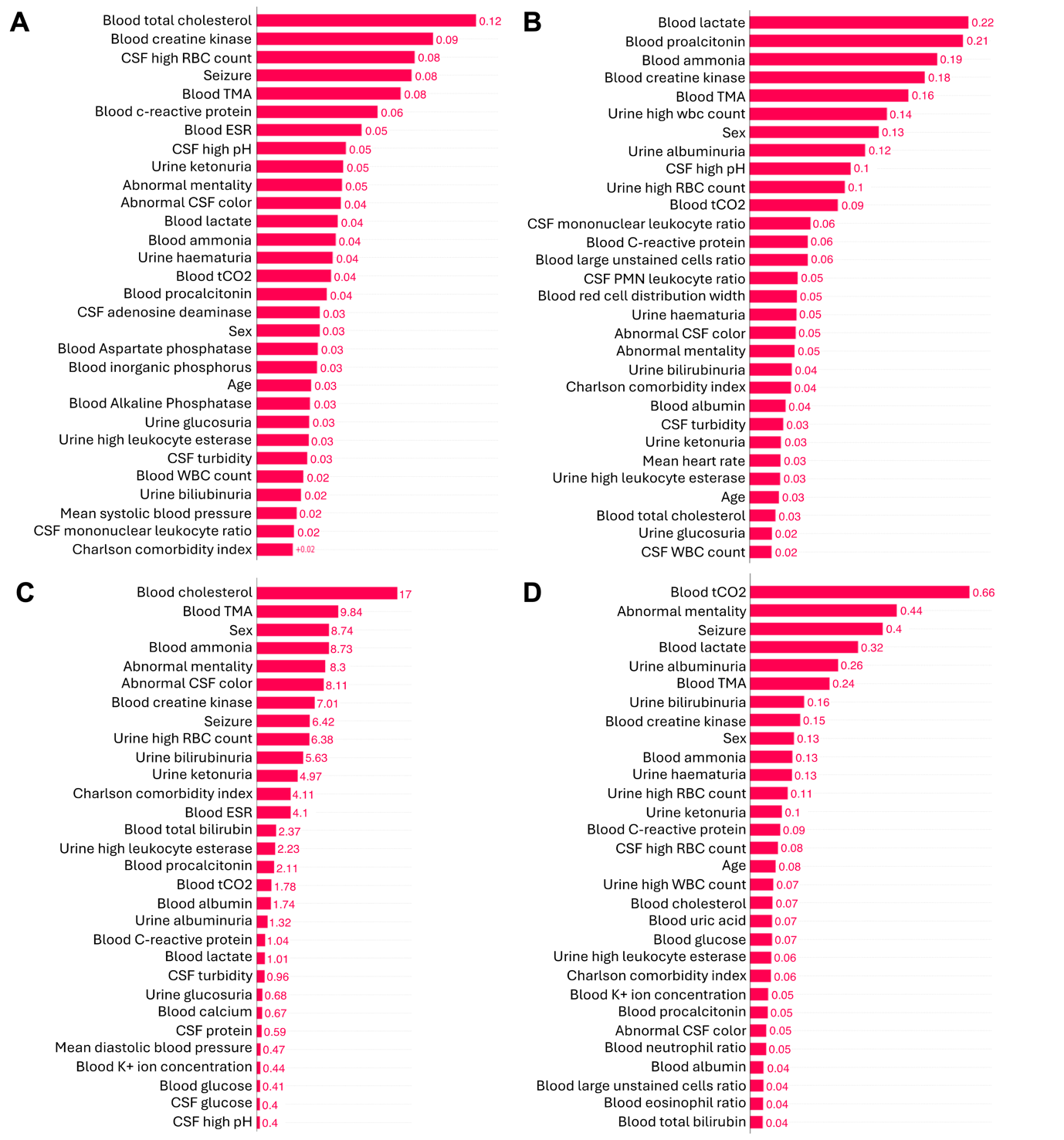
Supplementary Figure 4. The top 30 important clinical features selected for multimodal deep learning for each aetiology from unimodal model using clinical variables. We employed a batch-sampling strategy for SHAP estimation, selecting one-fifth of the test dataset at a time to compute SHAP values while balancing memory efficiency and estimation performance. This approach, combined with parallelized models, ensured local accuracy while managing substantial memory overhead. And the process was conducted using an internal dataset. (A) Autoimmune (n = 45, Image_n = 73) (B) Bacteria (n = 46, Image_n = 75) (C) Tuberculosis (n = 30, Image_n = 43) (D) Virus (n = 170, Image_n = 222).

* Abbreviations: RBC, red blood cell; TMA, thrombotic microangiopathy; ESR, erythrocyte sedimentation rate; CSF, cerebrospinal fluid; WBC, white blood cell; PMN, polymorpho-nuclear

Supplementary Figure 5. Top 20 variables crucially utilized in multimodal deep learning for each aetiology. We employed a batch-sampling strategy for SHAP estimation, selecting one-fifth of the test dataset at a time to compute SHAP values while balancing memory efficiency and estimation performance. This approach, combined with parallelized models, ensured local accuracy while managing substantial memory overhead. And this process was conducted using an external dataset. (A) Autoimmune (n = 29, Image_n = 78) (B) Bacteria (n = 8, Image_n = 15) (C) Tuberculosis (n = 3, Image_n = 5) and (D) Virus (n = 66, Image_n = 113).

* Abbreviations: CK, creatine kinase; CSF, cerebrospinal fluid; SBP, systolic blood pressure; ALP, alkaline phosphatase; ESR, erythrocyte sedimentation rate; VentralDC, ventral diencephalon; WBC, white blood cell; CRP, C-reactive protein; PMN, polymorphonuclear

Supplementary Figure 6. Uniform Manifold Approximation and Projection for Dimension Reduction (UMAP) plot based on the results of the Density-Based Spatial Clustering of Applications with Noise (DBSCAN) algorithm application to SHAP values. Clustering results of (A) autoimmune patients (n = 29, Image_n = 78) and (B) viral patients (n = 66, Image_n = 113) from the external dataset. A plot overlaying the modified Rankin Scale scores on the UMAP plot of (C) autoimmune and (D) viral patients.
